# Gastrointestinal Parasite Infections in Beef Cattle: A Comparative Study Between Conventional and Holistic Farms in Alentejo, Portugal

**DOI:** 10.3390/vetsci12100970

**Published:** 2025-10-10

**Authors:** Cátia Gomes, João Lozano, Mariana Louro, Lídia Gomes, José Luís Castro, Leonor Duarte, Feliciano do Carmo Reis, Luís Madeira de Carvalho

**Affiliations:** 1 CIISA—Centre of Interdisciplinary Research in Animal Health, Faculty of Veterinary Medicine, University of Lisbon, Avenida da Universidade Técnica, 1300-477 Lisbon, Portugal; jlozano@fmv.ulisboa.pt (J.L.);; 2 Associate Laboratory for Animal and Veterinary Sciences (AL4AnimalS), Avenida da Universidade Técnica, 1300-477 Lisbon, Portugal; 3 Laboratório de Parasitologia, Instituto Nacional de Investigação Agrária e Veterinária, 2780-157 Oeiras, Portugal; 4 Vetagromor Lda, Rua Sofia de Mello Breyner, 13C, 7050-165 Montemor-o-Novo, Portugalfelicianoreis@vetagromor.pt (F.d.C.R.)

**Keywords:** beef cattle, gastrointestinal parasites, holistic management, sustainability, Portugal

## Abstract

**Simple Summary:**

This study compared gastrointestinal (GI) parasite frequencies and shedding in beef cattle kept at conventional and holistic management farms in Alentejo, Portugal. Ninety-five faecal samples were analysed using parasitological techniques. Strongyles were the most frequent GI parasites (92.6%), followed by coccidia (38.9%) and *Strongyloides* spp. (10.5%). Although parasite frequency and egg shedding differed significantly between management systems, all animals had low infection levels. The results from this study suggest that holistic management may be an alternative and sustainable solution for parasite control in cattle farms.

**Abstract:**

Cattle are commonly affected by gastrointestinal (GI) parasites, which impact their welfare and productivity. Alternative management practices are currently being applied in several livestock farms worldwide to minimise or avoid the use of anthelmintic drugs on livestock in an unsustainable way. This study aimed to compare the presence of GI parasites between conventional and holistic management farms in Alentejo, Portugal. Between January and April 2024, a total of 95 faecal samples were collected from adult female beef cattle belonging to four conventional management farms and four holistic management farms. Parasitological diagnosis included the Mini-FLOTAC method, Willis flotation, natural sedimentation, modified Ziehl–Neelsen technique, and faecal cultures. Higher egg shedding levels were found at holistic management farms, and strongyles were the most frequent GI parasites (92.6%). In addition, *Oesophagostomum* spp. (37%) and *Trichostrongylus* spp. (32%) were the most frequent genera, although no significant differences were found between the two management systems. Moreover, animals from both groups presented low shedding values (<200 eggs per gram of faeces, EPG). These preliminary results allow us to suggest that adopting holistic management practices may be a sustainable solution for the control of GI parasite infections in cattle farms.

## 1. Introduction

Gastrointestinal (GI) parasite infections are still responsible for health and economic concerns in cattle farms worldwide. In addition, the problem concerning GI nematodes’ resistance to anthelmintics (AH) has increased along the years. This way, parasite control will be one of the most challenging medical procedures in future cattle clinical parasitology [1].

Currently, there are global concerns about climate change, planet sustainability, and the promotion and adoption of environmentally friendly practices. Consumers are now more worried about animal welfare, public health, and the environment, namely concerning the incorrect use of pharmaceuticals (e.g., antibiotics and antiparasitic drugs), which can lead to its accumulation in animal origin products [1]. Thus, the promotion of sustainable agricultural strategies that reduce or avoid pharmaceutical usage in farms is of upmost importance.

Holistic management (HM) is a regenerative farming system developed by Allan Savory, which combines ecological, economic, and social dimensions for decision-making. It is particularly recognised for its grazing management strategy, where livestock are managed at high densities on small plots for short periods, followed by long recovery intervals, thus mimicking the natural dynamics of wild herbivores, where trampling, dung, and urine contribute to soil fertility and pasture regeneration. By dividing grazing areas into smaller sections and rotating animals frequently, HM promotes biodiversity, improves pasture productivity, and supports sustainable livestock production. Recovery times are flexible and adjusted according to factors such as residual biomass, herd size, and seasonal or environmental conditions, ensuring both ecosystem resilience and long-term agricultural viability [2,3,4,5,6]. Farmers and researchers from various regions worldwide have reported numerous advantages associated with intensive pasture rotation. These include an increased biodiversity, landscape diversity, improved quality of life, enhanced soil, and livestock resilience and adaptability [4,7,8]. One of the key benefits of HM is the significant reduction or even elimination of the use of pesticides, herbicides, chemical fertilisers, and fire. This can be achieved since vegetation control is handled by the animals, substantial forest reserves are maintained, forest biodiversity is enhanced, feed costs are reduced, and the system becomes more capable of responding to climate changes and pest outbreaks [9].

A previous study conducted in South Africa highlighted HM’s potential to decrease both internal and external parasites in livestock [10]. Also, research performed in the UK reported that adopting grazing strategies such as simultaneous or alternate grazing with other herbivore species, can minimise parasite transmission, thus suggesting that these strategies can be incorporated into a holistic management framework to reduce the dependency from AH [11]. Fernández et al. [12] proposed that cattle grazing alongside pigs might lead to an effective control of ruminants’ parasitic infections, since pigs’ grazing habits, which disrupt cattle dung, result in the exposition of infective larvae to environmental factors that decrease their survival and consequent availability in the pasture.

Several studies performed in Europe have shown that good management practices, such as the usage of safe pastures, supplementary feeding, strategic turnout pasture management, and evasive grazing strategies without the use of AH, can be efficient in nematode control [13,14]. In addition, due to AH resistance, current control programmes can be costly to farmers and even unsustainable on a long-term basis [15]. In 2019, only 25% of organic farmers believed that the pharmaceutical industry would be able to develop improved treatments or vaccines before AH resistance becomes a problem [16]. To the best of our knowledge, in Portugal, there are no comparative data between conventional and holistic beef management farms concerning GI parasite frequency and shedding.

This study aimed to assess the presence of GI parasites and compare their frequencies and shedding between conventional and holistic management farms in Alentejo, Portugal, to test the hypothesis of finding significant differences for both variables between the two management systems, and thus check if the adoption of holistic strategies could be as effective as internal deworming protocols in GI parasite control.

## 2. Materials and Methods

### 2.1. Conventional and Holistic Management Farms

To support the collection of relevant data and distinguish management practices between conventional and holistic farms, a brief questionnaire was developed. The questionnaire was answered by the eight selected farms (four with holistic management and four with conventional management) and addressed the following aspects: (a) farm data—location and total area; (b) pasture data—type and area of pasture, division into paddocks, rotation frequency, species used in rotation, and entry of outside animals into the farm; (c) cattle herd data—number of breeding females and males, respective breeds, breeding season, and weaning age; (d) deworming practices—whether deworming is performed, frequency of administration, time from last administration, product used, and its duration; and (e) epidemiological data—parasitic diseases previously diagnosed and detection of clinical signs.

### 2.2. Sampling

This study was performed in cooperation with a farm animal veterinary clinic, VETAGROMOR. The clinic has four clients with holistically managed farms, which were included in our study. Conventionally managed farms were selected considering only their total number of breeding females; thus, both conventional and holistic farms had a similar number of samples. This way, a total of eight farms were included in this study, four with conventional management and four with holistic management. All were located in the Alentejo region (Portugal mainland).

**Figure 1:**
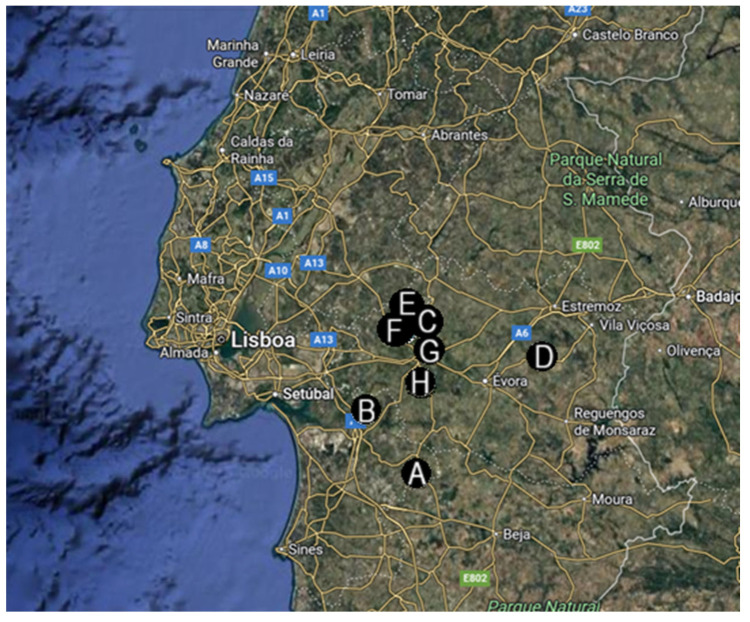
Geographic locations of the eight farms selected for this study. (**A**–**D**)—conventional management farms; (**E**–**H**)—holistic management farms; all farms were located in Portugal (mainland) (map retrieved from Google Earth, Google LLC).

Between January and April 2024, a total of 95 cattle faecal samples (45 samples from conventional management farms and 50 samples from holistic management farms) (Figure 1) were collected through rectal palpation and then placed in sterile cups. Sampling was initially planned to follow the general recommendation of collecting at least 10% of the breeding herd [17]. However, due to time and resource constraints, the protocol was adjusted: 10 samples were collected from herds of 100–200 animals, 15 from herds of 200–400 animals, and 20 from herds with more than 400 animals.

The sampling was performed in randomly chosen breeding female cattle (>24 months of age) and included the following breeds: Limousine, Aberdeen-Angus, Charolais, Mertolenga, and Alentejana. Faecal samples were transported in a cooling bag to the Laboratory of Parasitology and Parasitic Diseases of the Faculty of Veterinary Medicine, University of Lisbon (Lisbon, Portugal), and kept refrigerated at 4 °C for a maximum of three days, before being processed.

### 2.3. Coprological Techniques

#### 2.3.1. “3-in-1” Coprological Method

All samples were processed using the “3-in-1” protocol, which included the sequential use of the following techniques: Mini-FLOTAC, Willis flotation, and natural sedimentation. Briefly, for each faecal sample, five grams of faeces were mixed with 45 mL of saturated sucrose solution (relative density 1.2), using the Fill-FLOTAC device, and after careful homogenization the faecal suspension was transferred to a Mini-FLOTAC counting chamber. Parasite identification and counting were performed using an optical microscope (Olympus Iberia S.A.U., Barcelona, Spain), at 100× and 400× total magnifications, and with a detection limit of five eggs or oocysts per gram of faeces (EPG or OPG, respectively) [18,19].

The remaining suspension in the Fill-FLOTAC device was then used to perform the Willis flotation and natural sedimentation protocols. Briefly, the faecal suspension was poured into a 10 mL tube until forming a convex meniscus, to which a coverslip was added; after 10 min, the coverslip was removed and observed on a slide under the optical microscope, at 100× and 400× total magnifications, to identify coccidia oocysts and nematode and cestode eggs. Whenever the samples were negative for GI parasites in Mini-FLOTAC, but positive in the Willis flotation method, the overall result was considered as positive, with an egg or oocyst shedding of 5 EPG or OPG, respectively. After the flotation method, the supernatant was removed, and two drops of sediment were transferred to a slide and mixed with one drop of methylene blue. Visualisation was performed using an optical microscope, at 100× and 400× total magnifications, to identify trematode eggs. All parasite identifications were based on the morphological descriptions from Thienpont et al. [20] and Zajac and Conboy [21].

#### 2.3.2. *Cryptosporidium* spp. Oocysts Identification Using the Ziehl–Neelsen Modified (MZN) Technique

In this study, we performed the modified Ziehl–Neelsen (MZN) staining technique for *Cryptosporidium* spp. oocysts identification [22,23], using an optical microscope at 1000x total magnification (with immersion oil), to spot rose-reddish and nearly round oocysts, with a diameter of 2–6 μm [24].

### 2.4. Faecal Cultures

Faecal cultures were performed to morphologically identify nematode infective larvae, based on reference procedures from Madeira de Carvalho [25]. Briefly, for each farm, faecal cultures were performed using five pooled samples (2 g of each sample), with 10 g of stool in total; faeces were homogenised, and a donut form was established to allow oxygenation of the culture. Faecal cultures were maintained in the incubator for 14 days, at 26 °C and 70–80% relative humidity; after incubation, each cup was filled with tap water and rested in an inverted position in a Petri plate for 24 h, at room temperature and with moderate sunlight exposition, to allow L3 larvae to migrate from stool to water. Then, L3 larvae were collected in 10mL tubes and left for natural sedimentation. For each faecal culture, three slides were established with 100 µL of larval suspension and one drop of Lugol solution, and then observed under the optical microscope, at 100× and 400× total magnifications, for larval counts and morphological identification, based on the descriptions from Ueno and Gonçalves [26] and Wyk et al. [27].

### 2.5. Statistical Analysis

All data were stored using Microsoft^®^ Excel^®^ for Microsoft 365 (Microsoft Corporation, Redmond, WA, USA, 2025), which was also used to calculate parasite frequencies and generate charts.

Additionally, the software IBM^®^ SPSS^®^ Statistics version 27 (IBM Corporation, Armonk, NY, USA) was used to perform descriptive and inferential statistics. Firstly, the Kolmogorov–Smirnov Normality test was performed, and all EPG and OPG data were found to not be normally distributed (*p* < 0.001). Thus, inferential statistical analysis included the Mann–Whitney non-parametric test, which was used to compare two independent groups. Additionally, the Chi-square (X^2^) test and Fisher’s exact test were performed to assess parasite frequency differences between the two management systems (conventional and holistic). A significance level of *p* < 0.05 was considered for all tests.

## 3. Results

### 3.1. Conventional and Holistic Farms’ Management Conditions

The four conventional management farms included in this study were in the Torrão and São Martinho parishes (Setúbal district) (Figure 1A,B), and Ciborro and Freixo parishes (Évora district) (Figure 1C,D). The average farm area was 782.5 hectares, with irrigated pasture averaging 58 hectares. Herds averaged 168 breeding females and 5 breeding males, respectively, and belonged to the Limousin, Charolais, Angus, and Alentejana breeds. On average, producers rotated the animals every 30 days between pasture areas. The average weaning age was seven months. Regarding internal deworming protocols, two farms performed deworming procedures semi-annually, and two on an annual basis. All of them were using the same anthelmintic drug for an average of seven years: Moxidectin pour-on solution (Cydectin^®^ Pour-On) or an injectable solution combining Ivermectin and Clorsulon (Virbamec F^®^ Injectable Solution). None of the farms had previously reported cases of parasite infections. At the time of sample collection, the animals showed no clinical signs suggesting parasite infections.

The four holistically managed farms were located in the parishes of Lavre (Figure 1E,F) and Nossa Senhora da Vila, Nossa Senhora do Bispo, and Silveiras (Figure 1G,H), all in the municipality of Montemor-o-Novo. On average, the total area of the farms was 770 hectares, with an irrigated pasture averaging 71.25 hectares. The cattle herd averaged 262 breeding females and 8 breeding males, primarily of the Limousin, Angus, and Charolais breeds. Pasture rotation was performed twice daily or on intervals of up to five days. The average weaning age was six months. These farms reported the absence of internal deworming procedures for an average of eight years. There were no records of diagnosed parasitic diseases in any of the farms, and at the time of sample collection no animals showed clinical signs suggestive of parasite infections.

### 3.2. Parasitological Results

**Figure 2:**
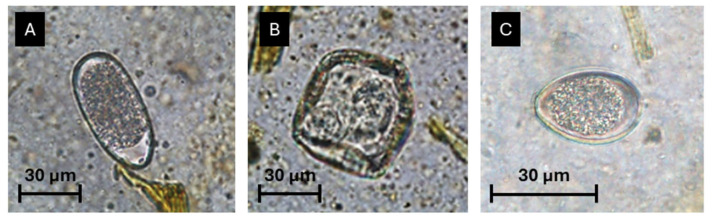
Helminth eggs and coccidia oocysts mostly identified using the “3-in-1” coprological procedure: (**A**) strongyle egg; (**B**) *Moniezia* spp. egg; (**C**) coccidia oocyst; scale bars—30 µm (original photos).

**Figure 3:**
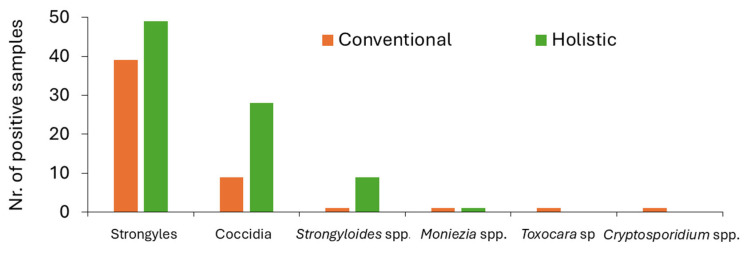
Number of positive samples for each parasite taxon in conventional (orange bars) and holistic (green bars) managements.

Within the total 95 faecal samples analysed, 94.7% were positive for the presence of GI parasitic forms using at least one of the coprological techniques previously mentioned. Moreover, 88 samples were found to be positive for GI strongyles (92.6%) (Figure 2), 37 samples positive for coccidia oocysts (38.9%), and 10 samples positive for *Strongyloides* spp. eggs (10.5%) (Figure 3). Furthermore, *Moniezia* spp. eggs were identified in two samples (2%), and *Toxocara vitulorum* eggs in one sample (1%) (Supplementary Materials).

**Figure 4:**
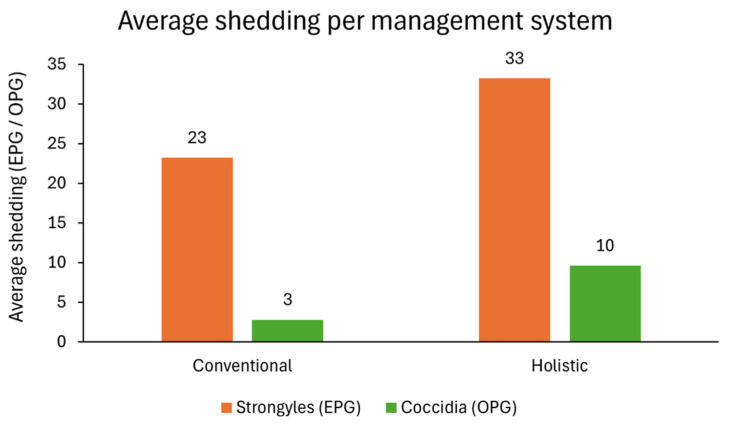
Strongyles EPG (orange bars) and coccidia OPG (green bars) average values for conventional and holistic management systems.

Overall, strongyle eggs were the most common GI parasitic forms, with a higher shedding being detected in faeces from holistic management animals. The same happened for coccidia oocysts, which were the second most common parasite found in quantitative methods, followed by *Strongyloides* spp. eggs, which presented a total of 55 EPG in cattle from holistic management farms and 5 EPG in conventional management farms. On average, animals excreted strongyle eggs in a range of 23–33 EPG, and coccidia oocysts ranged 3–10 OPG (Figure 4).

No trematode eggs were identified in this study, and only one sample (1%) was found to be positive for the presence of *Cryptosporidium* spp. oocysts.

Within the conventional management farms, strongyle eggs had the highest frequency (87%), followed by coccidia of the genus *Eimeria* (20%). In addition, eggs from the genera *Strongyloides* (2%), *Moniezia* (2%), and *Toxocara* (2%) were also identified, followed by *Cryptosporidium* spp. oocysts (2%) (Figure 3). Also, faecal egg counts ranged between 0 and 145 EPG for strongyle eggs, 0 and 50 OPG for coccidia oocysts, and between 0 and 5 for *Strongyloides* sp. eggs.

Regarding the holistic management farms, it was found that strongyle eggs were the most frequent (98%), followed by *Eimeria* spp. oocysts (56%). In addition, *Strongyloides* sp. eggs were revealed to be more frequent in this system, in comparison to conventional management (18%). Also, one sample was positive for *Moniezia* spp. eggs (2%). In this system, faecal egg counts ranged between 0 and 165 EPG for strongyle eggs, between 0 and 40 OPG for coccidia oocysts, and the highest *Strongyloides* sp. egg shedding totalled 10 EPG.

**Figure 5:**
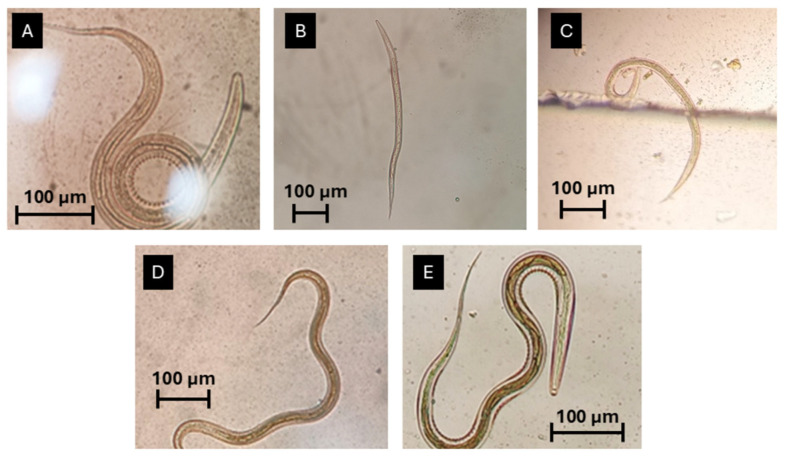
Third-stage larvae identified on faecal cultures: (**A**) *Oesophagostomum* sp. (the most prevalent genus); (**B**) *Trichostrongylus* sp.; (**C**) *Ostertagia* sp.; (**D**) *Haemonchus* sp.; (**E**) *Cooperia* sp.; scale bars—100 µm (original photos).

Overall, faecal cultures revealed that the most frequent genus was *Oesophagostomum* (37%), followed by *Trichostrongylus* (32%), *Ostertagia* (5%), *Haemonchus* (5%), and *Cooperia* (5%) (Figure 5) (Supplementary Materials). Regarding conventional management, the most frequently identified genus was *Trichostrongylus*, with a frequency of 33%, followed by *Oesophagostomum* (22%) and *Cooperia* (10%). Under holistic management, the most prevalent genus was *Oesophagostomum*, with a frequency of 50%. The second most prevalent genus was *Trichostrongylus* (30%), followed by *Ostertagia* (5%) and *Haemonchus* (5%).

Frequency and shedding data analysis revealed that there were significant differences for strongyle eggs, *Strongyloides* sp. Eggs, and coccidia oocysts between the two management systems (Table 1). However, no significant differences were found for larvae genera identified in both systems.

## 4. Discussion

The aim of this study was to assess the frequency and shedding associated with GI parasite infections in beef cattle produced in the Alentejo region, Portugal, and compare them between two management systems: conventional and holistic.

Overall results revealed that GI strongyles were the most frequent parasites, followed by *Eimeria* spp., *Strongyloides* sp., *Toxocara* spp. and *Moniezia* spp. Regarding faecal egg counts, both groups exhibited low levels of GI strongyle egg excretion. The highest EPG values were 145 EPG in the conventional management group and 165 EPG in the holistic management group, indicating only light infections (<200EPG) in both management systems [26]. Several studies previously performed in Portugal have identified GI strongyles as the most prevalent parasites detected in coprological surveys [28,29]. These findings corroborate our results for the conventional management group, although frequencies were higher and similar to those reported by Cardoso [30] and Borgsteede et al. [31]. The frequency of *Eimeria* spp. in conventionally managed cattle was in accordance with results from other studies on beef breeds [29,32,33]. In the case of *Strongyloides* sp., the frequency was higher in the holistic management group, although similar findings have also been reported [33].

In general, farms practicing holistic management showed higher frequencies for all parasite groups in comparison to conventional farms. A study conducted in Brazil found a frequency of 85.4% for GI strongyles in cattle grazing solely, being in accordance with our findings [34]. Another Brazilian study which compared organic and conventional grazing systems reported light to heavy parasite infections, with EPG levels reaching up to 1300 EPG in conventional management farms and 800 EPG in organic management farms, with significant seasonal differences observed only in February and May [35].

In Europe, organic management systems tend to show low to moderate levels of GI strongyle egg excretion. A study performed in Spain reported faecal egg counts in adult female cows as low as 0–30 EPG, even when there was no anthelmintic treatment [36]. Also, in Sweeden, a previous study in organic dairy farms identified that 98.6% of the samples had shedding levels below 500 EPG, and mostly between 50 and 300 EPG [13]. In addition, previous research on German organic farms reported strongyle frequencies less than 25%, besides coccidia and *Strongyloides* spp. infections [37]. Compared to these studies, our results revealed a higher frequency for all three parasitic forms, although the *Eimeria* spp. OPG was lower. Alternative grazing strategies have shown results similar to conventional management systems regarding the frequency and intensity of *Eimeria* spp. OPG and strongyle EPG, as observed in Poland and Germany [37,38], and in our study.

Notably, no trematode eggs, particularly of *Fasciola hepatica*, were detected in any of the analysed samples. This absence aligns with the low frequency of trematodes reported in other studies performed in Portugal and across Europe, regardless of the management system [28,33,37,38,39]. A possible explanation for this result may rely on the fact that the selected farms had mainly dryland grazing areas, and animals had no contact with irrigated pastures or marshy areas until the time of sample collection, and thus had no exposure to these parasites’ intermediate hosts (e.g., *Galba* spp.).

In this study, *Cryptosporidium* spp. were detected in only 1% of the samples, being a frequency consistent with previous reports in adult cattle [40] and notably lower than that observed in calves [22,41]. This likely reflects the reduced susceptibility of adult animals, although the use of the modified Ziehl–Neelsen technique, which has a lower sensitivity in comparison to immunofluorescence-based and molecular biology methods [42], may also have contributed to its underestimation.

This study did not find significant differences concerning the nematode genera identified between the two management groups. Overall, the most frequent were *Oesophagostomum* (37%) and *Trichostrongylus* (32%), which were identified in samples from both systems. While the nematode *Ostertagia ostertagi* has been reported as highly frequent in other studies [31,36], our findings are consistent with those from other studies conducted in both conventional and holistic management farms, which showed that this species is less represented in these beef cattle production systems [28,35,43,44]. This proximity of results between the selected groups can be explained by their geographical proximity, since the influence of environmental and climatic factors was similar for both systems.

Holistic management has been studied in Europe over the years, and it has revealed promising results in terms of sustainable parasite control. Studies in Sweden demonstrated that adequate parasite control may be achievable without the use of AH, with strategies like evasive grazing strategies, rotating grazing pastures between calves and cattle, and late turnout, allowing animals to maintain good zootechnical parameters and GI parasite infection intensities at low to moderate levels [13,14,37]. In our study, the holistic management group showed an overall low shedding level for GI strongyles, which may be explained by the implementation of sustainable grazing strategies, typical of this system.

## 5. Conclusions

The overall results from this study revealed that, despite the significant differences that were found for GI strongyles, *Strongyloides* spp. and *Eimeria* spp. between the management systems, cattle kept at holistic farms (where internal dewormings were not performed up to 10 years before our study) did not exhibit any clinical signs suggestive of parasite infection, while their faeces had a very low parasite shedding.

These findings suggest that alternative strategies to anthelmintic use could be a viable approach to parasite control in extensive cattle management, considering animal health, welfare, and productivity. Furthermore, routine coprological examinations should be performed to monitor subclinical infections at cattle farms. Future large-scale studies on this topic could address the influence of several biotic and abiotic factors (e.g., as animals’ age, breed, nutritional status, and climate) on parasite infection dynamics at conventional and holistic farms.

## Figures and Tables

**Table 1:** Data comparison between samples from conventional and holistic managements, and respective results concerning inferential statistical analysis.

Parasites	Management System		Differences Between Management Systems (*p*-Value)	
	Conventional	Holistic	EPG/OPG	Frequency
GI strongyles (eggs)	+	+	***p*** **= 0.005**	***p*** **= 0.041**
*Strongyloides* sp. (eggs)	+	+	***p*** **= 0.012**	***p*** **= 0.017**
*Eimeria* spp. (oocysts)	+	+	***p* ˂ 0.01**	**NA**
*Toxocara* spp. (eggs)	+	-	**NA**	*p* = 0.474
*Moniezia* spp. (eggs)	+	+	**NA**	*p* = 1.000
*Trichostrongylus* spp. (eggs)	+	+	*p* = 0.905	*p* = 1.000
*Ostertagia* sp. (larvae)	-	+	*p* = 0.720	*p* = 1.000
*Haemonchus* sp. (larvae)	-	+	*p* = 0.720	*p* = 1.000
*Cooperia* spp. (larvae)	+	-	*p* = 0.720	*p* = 0.474
*Oesophagostomum* sp. (larvae)	+	+	*p* = 0.278	*p* = 0.350
*Cryptosporidium* spp. (oocysts)	+	-	**NA**	*p* = 0.474
**Totals**	9	8		

+: at least one positive sample; -: all samples negative; NA: not applicable; significant differences in bold (*p* < 0.05)

## Data Availability

The original contributions presented in this study are included in the article/Supplementary Materials. Further inquiries can be directed to the corresponding author(s).

## Supplementary Materials

The following supporting information can be downloaded at https://www.mdpi.com/article/10.3390/vetsci12100970/s1. Supplementary File S1—global parasite shedding and frequency data for each management system.

## Abbreviations

The following abbreviations are used in this manuscript:

AH	Anthelmintics
EPG	Eggs per gram of faeces
GI	Gastrointestinal
HM	Holistic management
L3	Third-stage larvae
MZN	Modified Ziehl–Neelsen
OPG	Oocysts per gram of faeces

## References

[1] Forbes A. (2023). The future of farm animal parasitology. Vet. J..

[2] Savory A. (1983). The Savory Grazing Method or Holistic Resource Management. Rangelands.

[3] Savory A., Parsons S.D. (1980). The Savory Grazing Method. Rangelands.

[4] Mann C., Sherren K. (2018). Holistic Management and adaptive grazing: A trainers’ view. Sustainability.

[5] Briske D.D., Ash A.J., Derner J.D., Huntsinger L. (2014). Commentary: A critical assessment of the policy endorsement for holistic management. Agric. Syst..

[6] Hawkins H.J., Venter Z.S., Cramer M.D. (2022). A holistic view of Holistic Management: What do farm-scale, carbon, and social studies tell us?. Agric. Ecosyst. Environ..

[7] Stinner D.H., Stinner B.R., Martsolf E. (1997). Biodiversity as an organizing principle in agroecosystem management: Case studies of holistic resource management practitioners in the USA. Agric. Ecosyst. Environ..

[8] De Villiers A.C., Esler K.J., Knight A.T. (2014). Social processes promoting the adaptive capacity of rangeland managers to achieve resilience in the karoo, South Africa. J. Environ. Manag..

[9] Ferguson B.G. (2015). Experiencias de ganadería holística en Chiapas. Ecofronteras.

[10] Rapiya M., Hawkins H.-J., Muchenje V., Mupangwa J., Marufu M.C., Dzama K., Mapiye C. (2019). Rotational grazing approaches reduces external and internal parasite loads in cattle. Afr. J. Range Forage Sci..

[11] Fox N.J., Marion G., Davidson R.S., White P.C.L., Hutchings M.R. (2013). Modelling parasite transmission in a grazing system: The importance of host behaviour and immunity. PLoS ONE.

[12] Fernández S., Šarkunas M., Roepstorff A. (2001). Survival of infective *Ostertagia ostertagi* larvae on pasture plots under different simulated grazing conditions. Vet. Parasitol..

[13] Höglunda J., Svenssonb C., Hesslec A. (2001). A field survey on the status of internal parasites in calves on organic dairy farms in southwestern Sweden. Vet. Parasitol..

[14] Dimandera S.-O., Höglunda J., Ugglaa A., Spörndlyb E., Wallera P.J. (2003). Evaluation of gastrointestinal nematode parasite control strategies for first-season grazing cattle in Sweden. Vet. Parasitol..

[15] Morgan E.R., Charlier J., Hendrickx G., Biggeri A., Catalan D., Von Samson-Himmelstjerna G., Demeler J., Müller E., Van Dijk J., Kenyon F. (2013). Global Change and Helminth Infections in Grazing Ruminants in Europe: Impacts, Trends and Sustainable Solutions. Agriculture.

[16] Takeuchi-Storm N., Moakes S., Thüer S., Grovermann C., Verwer C., Verkaik J., Knubben-Schweizer G., Höglund J., Petkevičius S., Thamsborg S. (2019). Parasite control in organic cattle farming: Management and farmers’ perspectives from six European countries. Vet. Parasitol. Reg. Stud. Reports..

[17] Herd R.P. (1993). Control strategies for ruminant and equine parasites to counter resistance, encystment, and ecotoxicity in the USA. Vet. Parasitol..

[18] Cringoli G., Maurelli M.P., Levecke B., Bosco A., Vercruysse J., Utzinger J., Rinaldi L. (2017). The Mini-FLOTAC technique for the diagnosis of helminth and protozoan infections in humans and animals. Nat. Protoc..

[19] Barda B.D., Rinaldi L., Ianniello D., Zepherine H., Salvo F., Sadutshang T., Cringoli G., Clementi M., Albonico M. (2013). Mini-FLOTAC, an Innovative Direct Diagnostic Technique for Intestinal Parasitic Infections: Experience from the Field. PLoS Neglected Trop. Dis..

[20] Thienpont D., Rochette F., Vanparijs O.F.J. (2003). Worm eggs–Sheep and cattle. Diagnosing Helminthiasis Through Coprological Examination.

[21] Zajac A., Conboy G. (2012). Parasites of Domestic Animals–Ruminants and Camelids. Veterinary Clinical Parasitology, 8th edition.

[22] Pereira da Fonseca I.M.S. (2000). Contribuição Para o Estudo da Criptosporidiose Animal em Portugal: Caracterização Genética de Isolados de *Cryptosporidium parvum* de Origem Bovina. Ph.D. Thesis.

[23] Saramago J.M.G. (2019). Contribuição Para a Determinação de Infeção por *Cryptosporidium* spp. em Vitelos de Explorações de Carne das Sub-Regiões do Alentejo Central e Litoral. Master’s Thesis.

[24] Louro M., Bexiga R., Pereira da Fonseca I., Gomes J. (2024). Detection and molecular characterization of *Cryptosporidium* spp. in dairy calves in Lisbon and Tagus Valley, Portugal. Vet Parasitol Reg Stud Reports..

[25] Madeira de Carvalho L.M. (2002). Epidemiologia e Controlo da Estrongilidose em Diferentes Sistemas de Produção Equina em Portugal. Ph.D. Thesis.

[26] Ueno H., Gonçalves P.C. (1998). In Manual Para Diagnóstico das Helmintoses de Ruminantes.

[27] Wyk J.A.V., Cabaret J., Michael L.M. (2004). Morphological identification of nematode larvae of small ruminants and cattle simplified. Vet. Parasitol..

[28] Ramos J.C.D.S. (2013). Avaliação das Parasitoses Gastrointestinais em Bovinos de raça Brava Durante a Primavera e Verão. Master’s Thesis.

[29] Cachapa A.M.D. (2016). Avaliação da Eficiência do Programa de Controlo Antiparasitário Utilizado em Efetivos Bovinos de Carne na Região de Portalegre. Master’s Thesis.

[30] Cardoso J.M.G. (2010). Contribuição Para o Estudo do Parasitismo Gastrointestinal e Hepático em Bovinos de Carne em Regime Extensivo no Concelho de Odemira. Master’s Thesis.

[31] Borgsteede F.H.M., Tibben J., Cornelissen J.B.W.J., Agneessens J., Gaasenbeek P.H. (2000). Nematode parasites of adult dairy cattle in the Netherlands. Vet. Parasitol..

[32] Pimenta S., Malheiro A., Dores J., Dantas M., Mateus T.L. (2013). Diversidade de Parasitas Gastrointestinais em bovinos de raça Minhota–estudo preliminar. Agrotec Rev. Técnico-Científica Agrícola.

[33] Crespo M., Mariano P., Rosa F. (2013). In Parasitismo em Bovinos de raças de carne e Brava no Concelho de Coruche.

[34] Brito D.L., Dallago B.S.L., Louvandini H., Santos V.R.V.D., Torres S.E.F.D.A., Gomes E.F., do Amarante A.F.T., de Melo C.B., McManus C.M. (2013). Effect of alternate and simultaneous grazing on endoparasite infection in sheep and cattle. Rev. Bras. De Parasitol. Veterinária.

[35] Silva J.B., Fagundes G.M., Soares J.P.G., Fonseca A.H. (2013). Parasitism level by helminths and weight gain of calves kept in organic and conventional grazing. Braz. J. Vet. Res..

[36] Almería S., Uriarte J. (1999). Dynamics of pasture contamination by gastrointestinal nematodes of cattle under extensive management systems: Proposal for strategic control. Vet. Parasitol..

[37] Gillandt K., Stracke J., Hohnholz T., Waßmuth R., Kemper N. (2018). A field study on the prevalence of and risk factors for endoparasites in beef suckler cow herds in Germany. Agriculture.

[38] Pilarczyk B., Tomza-Marciniak A., Sablik P., Pilarczyk R. (2019). Parasites of the digestive tract in cows managed in alternative (organic and biodynamic) as well as conventional farms in West Pomerania. Ann. Parasitol..

[39] Malcata F.C.B. (2014). Prevalência e Controlo dos Parasitas Gastrointestinais em Explorações Bovinas Leiteiras em Portugal Continental. Master’s Thesis.

[40] Smith R.P., Clifton-Hadley F.A., Cheney T., Giles M. (2014). Prevalence and molecular typing of *Cryptosporidium* in dairy cattle in England and Wales and examination of potential on-farm transmission routes. Vet. Parasitol..

[41] de Barros S.V.A. (2015). Contribuição Para o estudo da Criptosporidiose em Vitelos de Explorações Leiteiras na Ilha Terceira, Açores. Master’s Thesis.

[42] Chalmers R.M., Campbell B.M., Crouch N., Charlett A., Davies A.P. (2011). Comparison of diagnostic sensitivity and specificity of seven Cryptosporidium assays used in the UK. J. Med. Microbiol..

[43] Oliveira C.C. (2018). Avaliação do Parasitismo Gastrointestinal em Bovinos de Carne em Sistema Extensivo e Semi-Extensivo. Master’s Thesis.

[44] Pilarczyk B., Balicka-Ramisz A., Kozak W., Ramisz A. (2009). Occurrence of endoparasites in heifers imported to Poland from the Netherlands (Short Communication). Arch. Anim. Breed..

